# Secondary Metabolites from Marine Sources with Potential Use as Leads for Anticancer Applications

**DOI:** 10.3390/molecules26144292

**Published:** 2021-07-15

**Authors:** Ana C. S. Veríssimo, Mário Pacheco, Artur M. S. Silva, Diana C. G. A. Pinto

**Affiliations:** 1 LAQV-REQUIMTE & Department of Chemistry, University of Aveiro, 3810-193 Aveiro, Portugal; carolinaana@ua.pt (A.C.S.V.); artur.silva@ua.pt (A.M.S.S.); 2 CESAM & Department of Biology, University of Aveiro, 3810-193 Aveiro, Portugal; mpacheco@ua.pt

**Keywords:** secondary metabolites, marine organisms, securamines, sterols, anticancer, cytotoxic activity

## Abstract

The development of novel anticancer agents is essential to finding new ways to treat this disease, one of the deadliest diseases. Some marine organisms have proved to be important producers of chemically active compounds with valuable bioactive properties, including anticancer. Thus, the ocean has proved to be a huge source of bioactive compounds, making the discovery and study of these compounds a growing area. In the last few years, several compounds of marine origin, which include algae, corals, and sea urchins, have been isolated, studied, and demonstrated to possess anticancer properties. These compounds, mainly from securamines and sterols families, have been tested for cytotoxic/antiproliferative activity in different cell lines. Bioactive compounds isolated from marine organisms in the past 5 years that have shown anticancer activity, emphasizing the ones that showed the highest cytotoxic activity, such as securamines H and I, cholest-3β,5α,6β-triol, (*E*)-24-methylcholest-22-ene-3β,5α,6β-triol, 24-methylenecholesta-3β,5α,6β-triol, and 24-methylcholesta-3β,5α,6β-triol, will be discussed in this review. These studies reveal the possibility of new compounds of marine origin being used as new therapeutic agents or as a source of inspiration to develop new therapeutic agents.

## 1. Introduction

Cancer is one of the most devastating and deadly diseases in the World. In 2020, more than 19 million new cases of cancer were reported globally, resulting in approximately 10 million deaths [1]. In terms of incidence and mortality, breast and lung cancers are the most common and are each responsible for more than 2 million diagnoses in 2020. The more frequent cancers in men are lung, prostate, and colorectum cancers, and lung cancer is responsible for more deaths (more than 1 million), followed by colorectum cancer. In women, breast cancer is the most frequent, followed by colorectum and lung cancers. In women, breast cancer has the highest mortality, with approximately 600,000 deaths [1]. Furthermore, it is estimated that in 2040 the number of new cancer cases in the globe will reach 28 million, with mortality beyond 16 million [2].

The high rates of cancer incidence have been reported with the increased risk of age and lifestyle changes, which are becoming less and less healthy [3,4]. In addition, it is necessary to consider the factors of cancer predisposition, such as tobacco, chemicals, radiation, and infectious organisms (external factors), as well as genetic predispositions and immune conditions (internal factors) [3]. Despite all the advances in cancer treatment [5,6], this is an area that is in constant development, always intending to achieve more effective therapies [7]. The cancer heterogeneity, associated with the characteristics and stage of the tumor [8], the resistance to anticancer agents [9], and the reduction in side effects related to treatments [10] are the most important obstacles to be overcome to achieve more effective therapy. This requires the development of new techniques/therapies and drugs. Cancer treatment can involve different therapies, such as surgery, chemotherapy, radiation therapy, and even immunotherapy [11,12]. Currently, the development of drugs for the treatment of cancer has been widely studied. One of the primary sources of these drugs are natural compounds from plants and marine organisms [13,14,15,16]. As well as compounds obtained from sources such as terrestrial plants, such as taxol, vincristine, and vinblastine [15,17], several natural products derived from marine sources have also been used in the prevention and treatment of various cancers, including leukemia, metastatic breast cancer, soft tissue sarcoma and ovarian cancer [18,19]. The ocean is home to around 250,000 species and is, thus, a great reservoir of life and biodiversity [20]. These marine organisms produce/synthesize several natural products. Synthesis of these marine natural products may be associated with the need on the part of these organisms to produce secondary metabolites as a defense tool to survive in extreme environments, such as temperature, salinity, pressure, and predators [21]. Marine flora has been used since ancient times for medicinal purposes globally, although its use is more common in oriental countries [22]. Bearing in mind that until recently, only a few marine organisms, such as microflora (bacteria, actinobacteria, cyanobacteria, and fungi), microalgae, macroalgae (seaweed), invertebrate animals, sponges, soft corals, sea fans, sea hares, nudibranchs, bryozoans, and tunicates have been investigated for cancer treatment [21,23,24,25,26], the ocean proves to be a significant and unknown source of bioactive compounds, making the discovery and study of these compounds an area of growth.

Thus, this review aims to highlight secondary metabolites isolated from marine organisms, which have been tested for cytotoxic activity in the last 5 years and have shown interesting IC_50_ values. Their activity level, chemical structure, and possibility of being used as drug leads will be discussed.

## 2. Secondary Metabolites from Marine Organisms with Cytotoxic Activity

In the last 5 years, several studies have been carried out with the aim of evaluating the bioactive properties of marine organisms. Several studies have tested the extracts and the isolated compounds from extracts of marine organisms for bioactivities [27,28,29,30,31,32,33,34,35,36,37,38,39,40,41,42,43,44,45,46,47,48], of which the cytotoxic activity [27,30,32,33,35,36,37,38,40,41,44,45,46,47,48] stands out. However, only in some studies was it possible to isolate the compounds and test them for cytotoxic activity [32,33,35,36,37,38,40,41]. The compounds isolated therein can be classified into the following families.

### 2.1. Securamines

**Figure 1:**
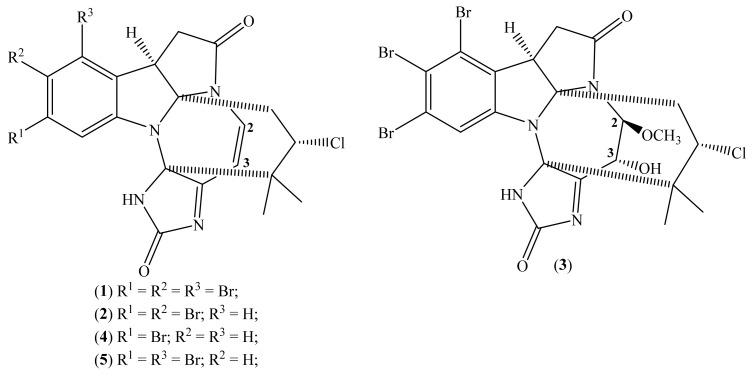
Chemical structure of securamine H (**1**), I (**2**), J (**3**), C (**4**) and E (**5**).

Securamines are halogenated indole-imidazole alkaloids characterized by a central tricyclic pyrroloindole core and a highly substituted imidazole ring linked via a modified isoprene subunit and a macrocyclic *cis*-enamide [49,50]. Examples of these compounds have been identified and isolated from *Securiflustra securifrons* (Pallas, 1766)*,* a marine bryozoan native to the North Sea (Figure 1) [33,50,51]. In addition, the securamines C (**4**) and E (**5**) have been isolated from *S. securifrons* in earlier studies [50,51]. In contrast, securamine H (**1**), I (**2**), and J (**3**) were isolated, from the same source, more recently [33]. In these works, all the derivatives were tested for different bioactivities [33,34], where cytotoxic activity stands out [33]. Table 1 describes the cytotoxic activity of these compounds against various human cancer cell lines, including A2058 (skin), HT-29 (colon), and MCF-7 (breast) and nonmalignant human MRC-5 lung fibroblasts.

Regarding evaluation of the cytotoxic activity of these compounds, the authors demonstrated that after 72 h, the compounds with the most significant cytotoxic activity were securamines H, I, C, and E (Figure 1; Table 1). In contrast, securamine J (**3**) did not demonstrate cytotoxic activity. Considering the structural differences and similarities of the compounds, it was concluded that the compounds with better activities had a double bond in C2=C3 and had two or more bromo as substituents in the aromatic ring. In contrast, methoxylation of C-2 and saturation of the C2=C3 appears to be detrimental to bioactivity [33].

The authors also evaluated the kinetics of cell death of the four cell lines for securamine H (**1**) case, for 4, 24, 48, and 72 h. This revealed that the cytotoxic activity of the tested compound is time dependent. The compound did not show cytotoxic activity in the first 4 h of exposure, but after 24 h, 48 h, and 72 h, the IC_50_ values decreased. In the A2058 cell line case, the IC_50_ decreased to 1.4 μM (Table 1). A similar pattern was found for the remaining cell lines, except for the nonmalignant MRC-5 cell line, which was significantly less affected after 24 h, with an IC_50_ > 10 μM. Regarding the absence of cytotoxic activity at the end of 4 h, it may indicate that the action of the compound, in the case of securamine H (**1**), is not associated with a rapid and nonspecific interaction with the cell membrane, leading to cell death. However, further studies are needed to uncover their mechanism of action [33]. Besides, suppose that the compounds will be considered leaders in discovering new anticancer drugs; in such a case, it is also essential that some modifications are necessary to improve their selectivity index (SI). The SI value, obtained through the ratio of IC_50_ for normal cells/IC_50_ for cancer cells, should be higher than 10 for the compound to be further evaluated [52], and, as can be seen in these compounds’ IC_50_ values (Table 1), they showed SI values below two.

### 2.2. Terpenoids

Several studies have shown that terpenoids can be molecules that are capable of helping to inhibit the growth of various cancers, opening new avenues for cancer treatment [53,54]. Recently, we demonstrated that seaweeds could be regarded as a source of several terpenoids with interesting anticancer activities, although many studies remain to be performed [55]. Herein, as stated above, we are focused on the last few years and looking at other marine resources. In that regard, Table 2 shows our choice of the most representative terpenoids isolated from marine organisms and their cytotoxic activity against cancer cell lines.

**Figure 2:**
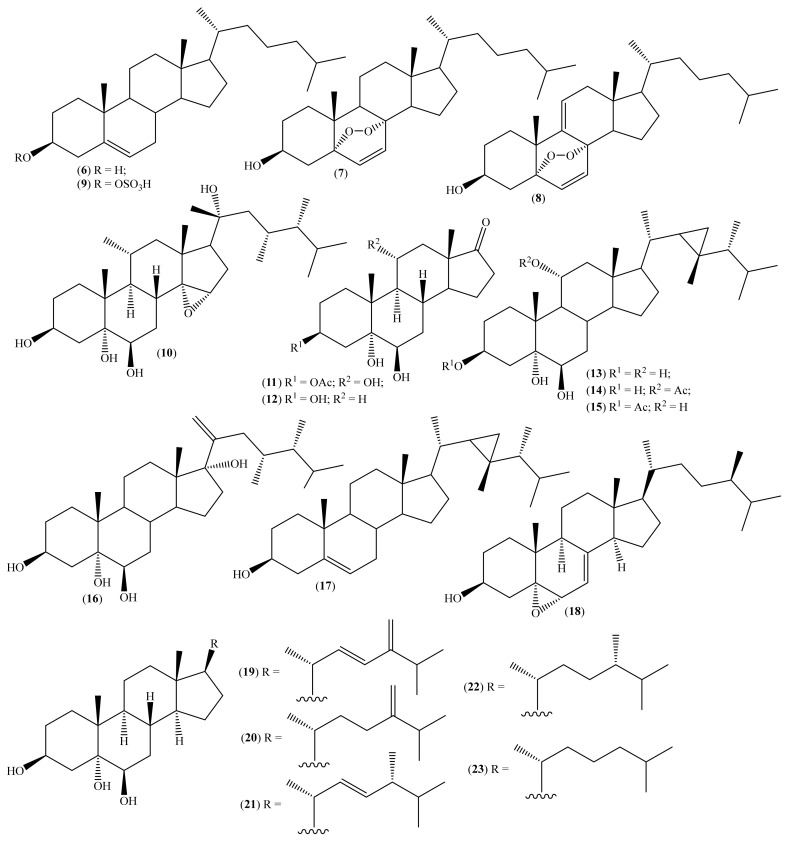
Chemical structure of steroid derivatives isolated from marine organisms.

Our first choice involves steroid derivatives (Figure 2) isolated from marine organisms such as the sea urchins *Diadema setosum* (Leske, 1778) and *Diadema savignyi* (Audouin, 1809) [35,56,57], and the corals *Heteroxenia fuscescens* (Ehrenberg, 1834), *Heteroxenia ghardaqensis* (Gohar, 1940), and *Lobophytum lobophytum*, including from *Sinularia* and *Sarcophyton* species [32,37,58,59,60,61,62,63], but also isolated from the sponges *Haliclona crassiloba* (de Laubenfels, 1950) and *Cliona copiosa* (Sarà, 1959) [64,65], and the marine invertebrate *Microcosmus vulgaris* (Heller, 1877) [36].

**Table 2:** Cytotoxicity of terpenoids isolated from marine organisms toward cancer cell lines.

Compound	Source ^a^	Cell Lines	IC_50_ (μM)	[Ref.] ^b^
Cholest-5-ene-3β-ol (**6**)	*D. setosum* *D. savignyi*	HeLa	>258 ^c^	[35,56,57]
5α,8α-Epidioxycholest-6-en-3β-ol (**7**)	*D. setosum* *D. savignyi*	HeLa	29.04 ± 6.58 ^c^	[35,56,57]
5α,8α-Epidioxycholest-6,9(11)-en-3β-ol (**8**)	*D. setosum*	HeLa	52.58 ± 15.24 ^c^	[35]
Cholest-5-ene-3β-ol-sulphate (**9**)	*D. setosum* *D. savignyi*	HeLa	>258 ^c^	[35,57]
Heterofuscesterol A (**10**)	*H. fuscescens*	MCF-7	72.57 ± 12.09 ^c^	[37]
		OVK-18	94.80 ± 7.94 ^c^	
Heterofuscesterol B (**11**)	*H. fuscescens*	MCF-7	>262.79 ^c^	[37]
		OVK-18	>262.79 ^c^	
3β,5α,6β-Trihydroxyandrosta-17-one (**12**)	*H. fuscescens*	MCF-7	>310.14 ^c^	[37]
		OVK-18	>310.14 ^c^	
Gorgost-3β,5α,6β,11α-tetrol (**13**)	*H. fuscescens* *H. crassiloba* *Sarcophyton* sp. *H*. *ghardaqensis*	MCF-7	>100	[32,59,60,61,64]
11α-Acetoxy-gorgost-3β,5α,6β-triol (**14**)	*H. fuscescens* *Sarcophyton* sp. *H. ghardaqensis*	MCF-7	33.2	[32,60,61]
3β-Acetoxy-gorgost-5α,6β,11α-triol (**15**)	*H. fuscescens*	MCF-7	>100	[32]
(*R*)-23-Methylergosta-20-ene-3β,5α,6β,17α-tetrol (**16**)	*H. fuscescens*	MCF-7	25.1	[32]
Gorgost-5(*E)*-ene-3β-ol (**17**)	*H. fuscescens* *H. ghardaqensis* *L. lobophytum*	MCF-7	>100	[32,61,62]
5α,6α-Epoxyergost-7-en-3β-ol (**18**)	*M. vulgaris*	HCT-16	>241.16 ^c^	[36]
(*E*)-24-Methylenecholestan-22-ene-3β,5α,6β-triol (**19**)	*Sinularia* sp.	HepG2	37.30	[58]
		HeLa	19.32	
24-Methylenecholesta-3β,5α,6β-triol (**20**)	*Sinularia* sp. *C. copiosa*	HepG2	13.36	[58,65]
		HeLa	16.55	
(*E*)-24-Methylcholest-22-ene-3β,5α,6β-triol (**21**)	*Sinularia* sp. *C. copiosa*	HepG2	13.66	[58,65]
		HeLa	18.31	
24-Methylcholesta-3β,5α,6β-triol (**22**)	*Sinularia* sp. *C. copiosa*	HepG2	12.40	[58,65]
		HeLa	8.82	
Cholest-3β,5α,6β-triol (**23**)	*Sinularia* sp. *C. copiosa*	HepG2	8.36	[58,65]
		HeLa	16.48	
(10*S*,11*S*)-Epoxyeleganediol (**24**)	*B. bifurcata*	MDA-MB-231	>310.09 ^c^	[40]
(14*R*)-14,15-Epoxyeleganediol (**25**)	*B. bifurcata*	MDA-MB-231	With 310.09 μM inhibited 78.8% ^c^	[40]
(11*R*)-11-Hydroxyeleganediol (**26**)	*B. bifurcata*	MDA-MB-231	>310.09 ^c^	[40]
(11*S*)-11-Hydroxyeleganediol (**27**)	*B. bifurcata*	MDA-MB-231	>312 ^c^	[40]
Eleganolone (**28**)	*B. bifurcata*	MDA-MB-231	42.70 ^c^	[40]
Dehydroderivative (**29**)	*B. bifurcata*	MDA-MB-231	109.30 ^c^	[40]
20-Hydroxygeranylgeraniol (**30**)	*B. bifurcata*	MDA-MB-231	32.63 ^c^	[40]
16-Hydroxygeranylgeraniol (**31**)	*B. bifurcata*	MDA-MB-231	47.31 ^c^	[40]
Heterofusceterpene A (**32**)	*H. fuscescens*	MCF-7	86.45 ± 12.00 ^c^	[37]
		OVK-18	141.64 ± 9.80 ^c^	
(6*R*,11*R*)-(−)-Furodysinin (**33**)	*H. infucata*	HeLa	>474.76 ^c^	[38]
Loliolide (**34**)	*B. bifurcata*	MDA-MB-231	>509.57 ^c^	[40]
Fucoxanthin analogue (**35**)	*B. bifurcata*	MDA-MB-231	>326.38 ^c^	[40]
Fucoxanthin (**36**)	*B. bifurcata*	MDA-MB-231	>151.77 ^c^	[40]
(1*R*,2*S*,4*R*,5*R*)-2-Bromo-4,5-dichloro-1-[(*E*)-2-chlorovinyl]-1,5-dimethylcyclohexane (**37**)	*P. capillaces*	HT-29	176.30 ± 27.08 ^c^	[41]
		LS174	155.30 ± 14.07 ^c^	

^a^ The Latin name herein used are the ones reported by the authors. ^b^ The list includes the first report concerning the isolation. ^c^ For the purpose of comparison, the values were converted from μg/mL to μM. IC_50_ = The concentration causing 50% inhibition of cell survival; HCT-16 = Colon cancer cell line; HeLa = Human cervical cancer cell line; HepG2 = Human liver cancer cell line; MCF-7 = Breast adenocarcinoma cell line; MDA-MB-231 = Breast cancer cell line; OVK-18 = Ovarian endometrioid carcinoma cell line.

From *D. setosum*, four steroids, compounds (**6**) to (**9**) (Figure 2; Table 2), were isolated and had their cytotoxic activity against human cervical cancer tested [35]. In this study, compound (**8**) was a new natural compound, isolated and identified for the first time [35]. Compound (**9**) was isolated for the first time from the species *D. setosum*; however, it was previously found in the species *D. savignyi* [57]. In the case of compounds (**6**) and (**7**), both had previously been isolated from the sea urchins *D. setosum* and *D. savignyi* [56,57].

Only compounds (**7**) and (**8**) demonstrated significant cytotoxic activity with IC_50_ 29.04 ± 6.58 μM and IC_50_ 52.58 ± 15.24 μM, respectively, when compared to the positive control used in the study, the 5-fluorouracil (IC_50_ 133.77 ± 28.44 μM). This suggests that the epidioxy group (Figure 2) greatly increases bioactivity. However, it seems that the number of double bonds in the tetracyclic structure of the epidioxy steroids also influences the compound activity towards human cervical cancer. Compound (**8**) presents an extra double bond (Figure 2), and its cytotoxic activity is lower than the one of compound (**7**) (Table 2) [35]. Although in this study, compound (**6**) does not demonstrate significant cytotoxic activity against human cervical cancer (Table 2), this compound has already shown, in previous studies, to be strongly cytotoxic to several cancer cell lines, including KB (human epidermoid carcinoma, IC_50_ 5.17 µM), FL (fibrillary sarcoma of the uterus, IC_50_ 10.16 µM), and Hep-2 (human hepatocellular carcinoma, IC_50_ 6.21 µM) [56]. Despite the high activity reported, it is necessary to have extra evaluations to establish the compounds’ selectivity towards the tumor cell lines.

Of the sterols isolated in *H. fuscescens* [32,37] (Table 2, Figure 2), compounds (**10**), (**11**), (**15**), and (**16**) are new natural sterols. Compound (**12**) was isolated for the first time from natural sources, although it was previously obtained by synthesis [66]. Compounds (**13**), (**14**), and (**17**) have already been identified in other marine organisms, such as corals [59,60,61,62,63] and sponges [64].

The cytotoxic activity of compounds (**10**), (**11**), and (**12**) was evaluated against cell lines MCF-7 and OVK-18, with vinblastine as a positive control. Of these compounds, compound (**10**) showed the best cytotoxic activity against cell lines MCF-7 (IC_50_ 72.57 ± 12.09 μM) and OVK-18 (IC_50_ 94.80 ± 7.94 μM) [37]. These IC_50_ values are lower than the control used in the study, vinblastine, for which the IC_50_ values for MCF-7 and OVK-18 were 65.23 ± 5.59 μM and 43.65 μM, respectively [37]. Compounds (**13**) to (**17**) were tested only against the MCF-7 cell line, with 5-fluorouracil as a positive control [32]. Only compound (**14**) and compound (**16**) showed relevant cytotoxicity against the MCF-7 cell line, with an IC_50_ of 33.2 μM and 25.1 μM, respectively, when compared to the control (IC_50_ = 18.7 μM) [32]. The results obtained suggest that the substitution of the side chain by a carbonyl group greatly reduces the cytotoxic activity of sterol [37]. Furthermore, even though the presence of the 3β,5α,6β-trihydroxy groups in the steroid skeleton is essential for cytotoxic activity, the presence of the 11α-acetoxy group markedly increases the cytotoxic activity of compounds with gorgosteroid skeletons [32]. Abdelkarem et al. [32,37] detailed the cytotoxic evaluation against breast adenocarcinoma ovarian endometrioid carcinoma cell lines; however, some studies involving mechanisms of action as well as evaluations of the compound’s selectivity are essential for further research [67].

The ascidian *M. vulgaris* is known to produce toxins but also, in some countries, is considered a food delicacy [68]; therefore, Konuklugil et al. performed a phytochemical study and isolated the sterol 5α,6α-epoxyergost-7-en-3β-ol (**18**) (Figure 2). Simultaneously, the compound was also tested against HCT-16 [36]. However, its cytotoxicity against the study cell line was not significant, and it had much a higher IC_50_ (5000× greater) than the IC_50_ of the positive control (Docetaxel) [36].

The *Sinularia* genus is a large one, with more than 90 species, some described more recently, and many of these soft corals are used in home aquariums [69,70]. Sun et al. isolated from *Sinularia* sp. a new trihydroxysterol, the (*E*)-24-methylenecholestan-22-ene-3β,5α,6β-triol (**19**) (Figure 2), and four known trihydroxysterols (**20**–**23**) (Figure 2) [58]. These last derivatives were previously isolated from *Sinularia* sp. [63] but also from the sponge *C. copiosa* [65].

Sun et al. also evaluated the antitumor activity of the sterol derivatives against HepG2 and HeLa (Table 2). All trihydroxysterols tested exhibited moderate to significant cytotoxicity against the two cell lines despite having different levels of efficacy. Compound (**19**) was the one with the least biological effect, exhibiting almost three times less toxicity than the other compounds against the HepG2 cell line. Compounds (**22**), 24-methylcholesta-3β,5α,6β-triol, and (**23**), cholest-3β,5α,6β-triol (Figure 2), demonstrated significant potential against cell lines. Looking at the structure of the compounds, it appears that the compounds with the highest cytotoxicity, (**22**) and (**23**), do not have double bonds in the side chain.

In contrast, compound (**19**) has two double bonds, and compounds (**20**) and (**21**) have one double bond (Figure 2). This suggests that the presence of double bonds in the side chains significantly influenced the biological effect studied, demonstrating that more double bonds in the side chain result in less cytotoxicity, regardless of the double bond position [58]. Although some of the reported IC_50_ values are interesting, there is no reference to the IC_50_ value for the positive control used in the study, which, in our opinion, reduces the value of the reported results.

Bearing in mind that significant cytotoxic activity may be considered for compounds having an IC_50_ below 40 μM, it can be perceived from Table 2 that sterols may be regarded as potential drug leads for developing new anticancer drugs. In fact, eight derivatives showed significant cytotoxic activity towards the studied cancer cell lines (Table 2).

**Figure 3:**
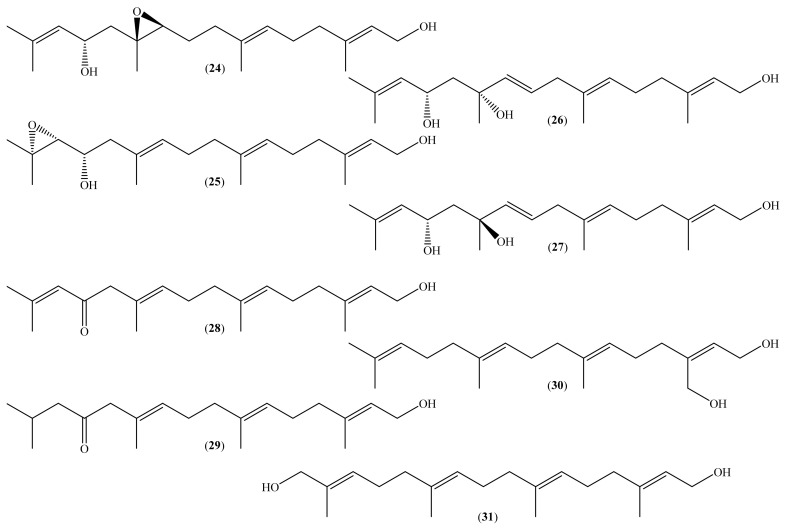
Chemical structure of linear diterpenes isolated from marine organisms.

Linear diterpenes are common in brown algae, including *Bifurcaria bifurcata* (R. Ross, 1958) [71], and are also recognized for their biological activities [72]. Recently, Smyrniotopoulos et al. isolated, from *B. bifurcata*, eight linear diterpenes (**24**–**31**) (Figure 3) and tested their cytotoxic activity against the human breast cancer line MDA-MB-231 (Table 2) [40]. Four of the isolated linear diterpenes (**28**–**31**) (Figure 3) demonstrated moderate activity, with IC_50_ values of 42.70, 109.30, 32.63, and 47.31 μM, respectively (Table 2). However, compound (**25**) also inhibited the growth of MDA-MB-231 cells by 78.8% to a concentration of 310.09 μM (Table 2). According to the authors, it was impossible to determine an IC_50_ value due to the small amounts of the compound available [40]. The other tested linear diterpenes have no cytotoxic activity (IC_50_ > 300 μM), and the IC_50_ value of the used positive control (5-fluorouracil) was not reported [40]. It seems evident that the tested compounds are closer to having moderate activity, with IC_50_ values between 40 μM and 110 μM, than significant activity. However, more studies are necessary to disclose the full potential of these terpenoids.

**Figure 4:**
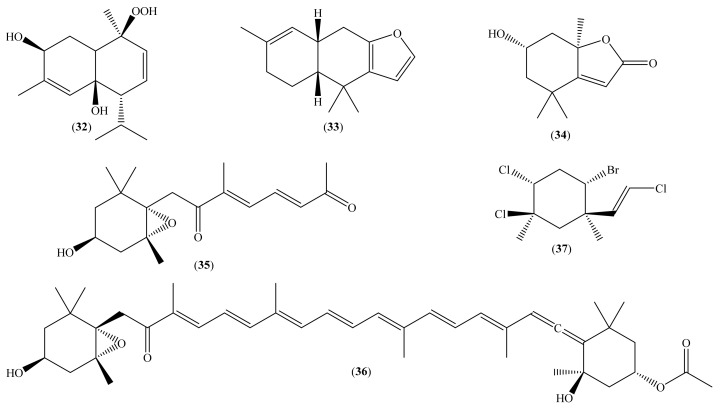
Chemical structure of other terpenoids isolated from marine organisms.

Other terpenoids, specific compounds (**32**–**37**) (Figure 4), were recently isolated from marine resources and had their cytotoxic activity evaluated (Table 2). Although most do not present cytotoxic activity, it should be highlighted that this is valid for the reported assays. For example, fucoxanthin (**36**) (Figure 4) is recognized for its many established activities, including anticancer [55,73], and it did not inhibit the growth of the breast cancer cell line MDA-MB-231 (Table 2).

Compound (**32**) (Figure 4), a sesquiterpene isolated from the coral *H. fuscescens*, showed moderate cytotoxic activity against MCF-7 (IC_50_ 86.45 ± 12.00 μM) (Table 2), with an IC_50_ that is half that reported for the positive control (vinblastine), and against OVK-18 (IC_50_ 141.64 ± 9.80 μM) [37]. In the case of compound (**33**) (Figure 4), isolated from the sea slug *H. infucata*, the IC_50_ against the human cervical cancer cell line HeLa is 474.76 μM, a very high value, especially when compared to the positive control, doxorubicin (IC_50_ 4.23 μM) [38].

Finally, we have the example of a polyhalogenated monoterpene compound (**37**) (Figure 4), isolated from algae *Pterocladiella capillaces* (S. G. Gmelin) (Santelices & Hommersand, 1997) [41,71]. This compound was evaluated for its inhibitory effect on the viability of two human colorectal adenocarcinoma cell lines, HT29 and LS174, and relevant studies on its cytotoxic action mechanism were also performed. For example, it was demonstrated that its effect is related to the activation of the ERK-1/-2, Akt, and NF-κβ pathways, contributing to the inhibition of the viability of the studied cell lines. Furthermore, it was also found that the compound induces cell cycle arrest in G2/M; this is associated with a decrease in the phosphorylated forms of the anti-tumor transcription factor p53, retinoblastoma protein (Rb), cdc2, and chkp2. In addition, it was shown that the compound triggers caspase-dependent apoptosis by activating caspase-3 and cleavage of poly (ADP-ribose) polymerase (PARP), and that it significantly increased the level of TRADD protein, a protein associated with the receptor for cell death [41].

### 2.3. Other Secondary Metabolites

**Figure 5:**
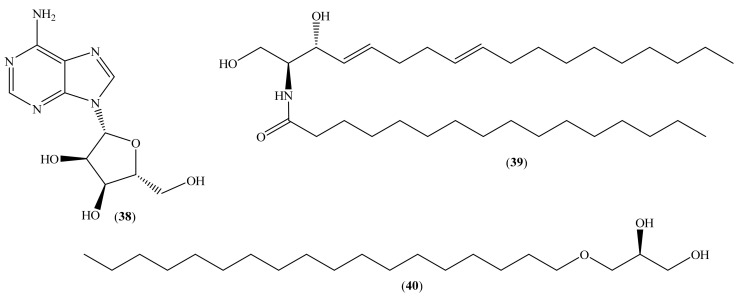
Chemical structure of compounds (**38**–**40**).

In addition to the compounds mentioned before, three other compounds were also isolated and identified (Figure 5): the nucleoside (**38**) [35], the ceramide (**39**) [37], and the glycerol derivative (**40**) [32].

Adenosine (**38**) [35] was previously isolated from marine organisms, the sponge *Tethya aurantium* (Pallas, 1766) [74], and the tunicate *Eudistoma laysani* (Sluiter, 1900) [75]. The ceramide (2*S*,3*R*,4*E*,8*E*)-2-amino-*N*-hexadecanoyl-4,8-octadiene-1,3-diol (**39**) was isolated and identified for the first time in the horn coral *Acabaria undulata* (Kükenthal, 1908) [76], but was also isolated from the sea urchin *D. setosum* [56]. Finally, the glycerol derivative (**40**) was isolated and identified in different marine organisms, such as corals and sponges [77]. However, the cytotoxic activity of these three compounds was recently evaluated [32,35,37].

Adenosine (**38**) was evaluated against the human cervical cancer cell line [35]; ceramide (**39**) was assessed against the breast adenocarcinoma cell line MCF-7 and ovarian endometrioid carcinoma cell line OVK-18 [37]. In addition, the glycerol derivative (**40**) was also evaluated against the MCF-7 cell line [32]. The reported results showed that against the tested cell lines, the compounds have no cytotoxic activity, and the IC_50_ values are above 180 μM [32,35,37].

## 3. Conclusions and Future Perspectives

This review discussed the cytotoxic activity of 40 secondary metabolites isolated from different marine organisms (from algae, corals, sea urchins, and marine invertebrates). When possible, mechanisms of action and structure/activity relationship were also discussed. Of the compounds isolated in the last 5 years, and discussed in this review, 13 have significant cytotoxic activity, with IC_50_ < 40 μM, and seven have moderate activity (40 μM < IC_50_ < 110 μM). The compounds reported in the greatest number are terpenoids, followed by securamines.

Securamines proved to be a promising class of compounds since four of the five securamines reported here have IC_50_ values < 30 μM. The ones that stand out are securamine H (**1**) and securamine I (**2**), with IC_50_ < 6 μM for the human melanoma cell line (A2058), human melanoma cell line (HT-29), breast adenocarcinoma cell line (MCF-7) and nonmalignant lung fibroblast cell line (MRC-5). In terpenoids, sterols isolated from *Sinularia* sp., compounds (**19**–**23**) are of note. All compounds isolated from this coral genus showed moderate to significant activity against the human liver cancer cell line (HepG2) and human cervical cancer cell line (HeLa). However, 24-methylenecholesta-3β,5α,6β-triol (**20**), (*E*)-24-methylcholest-22-ene-3β,5α,6β-triol (**21**), 24-methylcholesta-3β,5α,6β-triol (**22**) and cholest-3β,5α,6β-triol (**23**), with IC_50_ < 19 μM, are the most promising terpenoids. Thus, the compounds that showed moderate to significant activity in terpenoids failure are mainly sterols. We highlight that only compound (**37**) reveals studies on its cytotoxic action mechanism and has shown promising results. Additionally, only the evaluations of the securamines involved selectivity index studies.

In the treatment of cancer, more and more medicines/leads from natural products are used. With this review, we tried to show the compounds identified in recent years, which can contribute to improving this area. With the results compiled here, the role of molecules such as securamines and sterols as potential chemotherapeutic agents against various cell lines is evaluated. In addition, and considering the structure/activity relationships discussed here, these molecules appear to be possible natural product leads. However, further research is needed to understand structure/activity relationships and to assess their cytotoxicity in non-tumor cells. Furthermore, selectivity studies are also required to determine which cell lines are most affected by the most promising metabolites. In addition, the most promising metabolites should be evaluated in in-vivo studies, and their mechanism of action should also be disclosed.

## Figures and Tables

**Table 1:** Cytotoxicity of securamines, isolated from the marine organism *S. securifrons* ^a^, toward cancer cell lines and the nonmalignant cell line MRC-5 [33].

Cell Lines Tested	IC_50_ ^b^ (μM)				
	Securamine H (1)	Securamine I (2)	Securamine J (3)	Securamine C (4)	Securamine E (5)
A2058	1.4	2.7	>50	20	6.7
HT-29	1.9	2.5		21	10
MCF-7	2.1	2.4		23	8.5
MRC-5	2.7	5.3		30	9.6

^a^ In this table are indicated the Latin names used by the authors; ^b^ IC_50_ values refer to the concentration (μM) using 50% inhibition of cell survival; A2058 = Human melanoma cell line; HT-29 = Colon adenocarcinoma cell line; MCF-7 = Breast adenocarcinoma cell line; MRC-5 = Nonmalignant lung fibroblast cell line.
